# A Fast and Efficient Single-stranded Genomic Library Preparation Method Optimized for Ancient DNA

**DOI:** 10.1093/jhered/esab012

**Published:** 2021-03-25

**Authors:** Joshua D Kapp, Richard E Green, Beth Shapiro

**Affiliations:** 1 Department of Ecology and Evolutionary Biology, University of California Santa Cruz, Santa Cruz, CA; 2 Department of Biomolecular Engineering, University of California Santa Cruz, Santa Cruz, CA; 3 Genomics Institute, University of California Santa Cruz, Santa Cruz, CA; 4 Howard Hughes Medical Institute, University of California Santa Cruz, Santa Cruz, CA

**Keywords:** next generation sequencing, degraded DNA, genomics

## Abstract

We present a protocol to prepare extracted DNA for sequencing on the Illumina sequencing platform that has been optimized for ancient and degraded DNA. Our approach, the Santa Cruz Reaction or SCR, uses directional splinted ligation of Illumina’s P5 and P7 adapters to convert natively single-stranded DNA and heat denatured double-stranded DNA into sequencing libraries in a single enzymatic reaction. To demonstrate its efficacy in converting degraded DNA molecules, we prepare 5 ancient DNA extracts into sequencing libraries using the SCR and 2 of the most commonly used approaches for preparing degraded DNA for sequencing: BEST, which targets and converts double-stranded DNA, and ssDNA2.0, which targets and converts single-stranded DNA. We then compare the efficiency with which each approach recovers unique molecules, or library complexity, given a standard amount of DNA input. We find that the SCR consistently outperforms the BEST protocol in recovering unique molecules and, despite its relative simplicity to perform and low cost per library, has similar performance to ssDNA2.0 across a wide range of DNA inputs. The SCR is a cost- and time-efficient approach that minimizes the loss of unique molecules and makes accessible a taxonomically, geographically, and a temporally broader sample of preserved remains for genomic analysis.

Ancient DNA, or DNA that persists after organismal death, can provide unique insights into evolutionary history. Over the last 3 decades, ancient DNA has been used to place extinct taxa in phylogenetic trees (Shapiro et al. 2002; Bunce et al. 2005, 2009; Orlando et al. 2008) to reconstruct dynamics of extinct populations and communities (Shapiro et al. 2004; Stiller et al. 2010; Lorenzen et al. 2011), and to reveal past ecological changes such as extinction events or turnovers in community composition (Graham et al. 2016; Pedersen et al. 2016). With the advent of Next Generation Sequencing (NGS) technologies and consequent ability to sequence much shorter DNA fragments, the temporal and geographic scope of ancient DNA has expanded (Orlando et al. 2013; Brace et al. 2016; Meyer et al. 2017) and it has become feasible to sequence entire genomes from extinct species, which has facilitated the reconstruction of fine-scale evolutionary histories for many species, including our own (Green et al. 2010; Lazaridis et al. 2014). Despite these successes, the field remains limited by challenges in efficiently recovering short fragments of often damaged DNA from preserved biological remains, all of which are ultimately finite resources (Shapiro and Hofreiter 2014; Green and Speller 2017).

After organismal death, DNA damage accumulates in every cell via environmental, enzymatic, and chemical mechanisms (Dabney et al. 2013b). Most commonly, DNA strands become fragmented, likely via the accumulation of single-stranded breaks by hydrolytic depurination followed by β elimination (Lindahl 1993; Briggs et al. 2007). Additionally, the resulting fragments become chemically altered via deamination of cytosines to uracils at strand termini (Hofreiter et al. 2001; Briggs et al. 2007). The rate of depurination is influenced by temperature (Lindahl and Nyberg 1972), which means that preservation is environment-dependent, with the slowest degradation occurring in cold, temperature stable, and dry environments (Smith et al. 2003). Following collection from the environment, formalin-fixation (Van Beers et al. 2006; Stiller et al. 2016) or storage in warm or moist environments can also promote degradation. As a consequence of accumulating DNA damage, the number of recoverable molecules decays over time and, consequently, so does the sample’s utility for ancient DNA analysis.

Over the last 3 decades, methods have been developed that optimize recovery and processing of the short and damaged DNA fragments preserved in organismal remains. Ancient DNA optimized extraction protocols, for example, use in-solution silica binding (Rohland and Hofreiter 2007), silica spin columns (Dabney et al. 2013a), or magnetic beads (Rohland et al. 2018), to retain short DNA molecules. Approaches have also been developed that increase the quality and proportion of extracted authentically ancient molecules, for example, by repairing or excising DNA damage (Briggs et al. 2009; Mouttham et al. 2015; Rohland et al. 2015), by enzymatic depletion of microbial DNA (Green et al. 2010), or by enriching for damaged (and therefore authentically ancient) molecules (Gansauge and Meyer 2014).

After extraction, ancient DNA molecules must be converted into sequencing libraries via the addition of platform-specific DNA sequencing adapters at the ends of each molecule. Most commercially available library preparation approaches for Illumina sequencing perform poorly with damaged and degraded DNA (Stiller et al. 2016; Gansauge et al. 2017). For example, the commonly used New England Biolabs (NEB, Ipswich, MA) Ultra II DNA library preparation kit discards short fragments during clean-up steps, cuts uracil bases (which form naturally in ancient extracts via depurination) with the USER enzyme cocktail, and uses a non-uracil tolerant polymerase, all of which will reduce the recovery of ancient DNA molecules. These challenges have led to the development of several ancient DNA-specific approaches to library preparation.

The most commonly used library preparation methods optimized for degraded DNA process double-stranded DNA (dsDNA). The Meyer and Kircher (MK) approach (Meyer and Kircher 2010), for example, includes an end-repair step that fills in or chews back bases present as single-stranded overhangs to create blunt-ended molecules onto which the sequencing adapters can be ligated. However, the blunt-end ligation of the 2 sequencing adapters is non-directional, which means that either of the 2 adapters can be added to each end of the molecule. Because only molecules that have one of each adapter in the correct orientation can be sequenced, half of the molecules are lost due to incompatible adapter combinations. Additionally, MK requires 3 purification steps prior to amplification, all of which lose unique molecules. A more recently developed double-stranded DNA library preparation protocol, BEST (Carøe et al. 2017) (Figure 1A), is performed in a single tube, using heat denaturation rather than purification between reaction steps. BEST has been shown to yield higher complexity libraries compared to other double-stranded library protocols (Carøe et al. 2017), which may be partly explained by the reduction in unique molecule loss from limiting the number of purification steps. However, like MK, BEST also uses blunt-end repair and non-directional ligation scheme to add the sequencing adapters to double-stranded DNA molecules.

**Figure 1.**
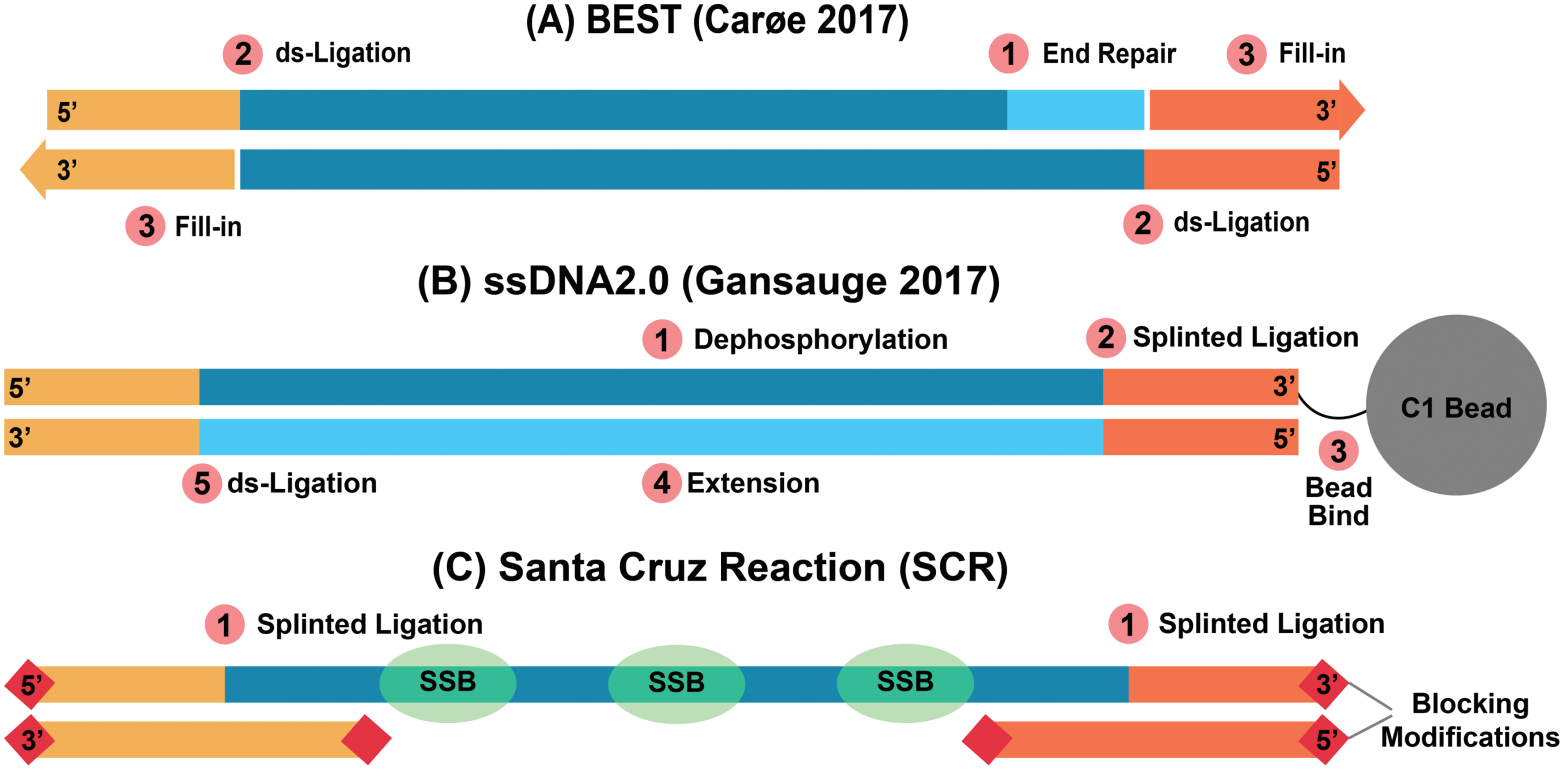
Key steps of the 3 library preparation methods compared here. (**A**) The BEST protocol begins with an input DNA end-repair step where 3′ overhangs are digested and 5′ overhangs are filled in (1). Then, double-stranded adapters are ligated to the 5′ ends of the input DNA (2), followed by adapter fill-in with a polymerase extension step, which initiates at the 3′ nick present among adapter ligated input DNA molecules (3). (**B**) ssDNA2.0 begins with the dephosphorylation of the input DNA (1), then a biotinylated adapter is ligated via splinted ligation to the 3′ end of the input DNA (2), which is then bound to streptavidin beads (3). After annealing an extension primer, a second strand is synthesized (4), and then the 5′ end of a double-stranded adapter is ligated to the 3′ end of the synthesized strand (5). (**C**) The Santa Cruz Reaction simultaneously ligates Illumina’s P5 and P7 adapter using splinted ligation (1).

Some ancient-DNA-specific library preparation approaches the target and converts single-stranded DNA (ssDNA) rather than dsDNA (Gansauge and Meyer 2013; Gansauge et al. 2017). Single-stranded library preparation methods begin with a denaturation step in which all DNA molecules in the extract are converted to single-stranded form. This allows conversion of DNA that is preserved in a single-stranded state as well as separate conversion of both strands of DNA preserved in a double-stranded state. When working with degraded DNA, ssDNA library preparation methods are more efficient, converting more DNA fragments into adapter-ligated form, compared to double-stranded approaches (Bennett et al. 2014; Wales et al. 2015; Gansauge et al. 2017). Additionally, some ssDNA library preparation methods leave the ends of DNA molecules unaltered, which makes it possible to explore patterns of stranded DNA fragmentation in aDNA extracts (Bokelmann et al. 2020).

Although ssDNA library preparation approaches improve DNA library conversion compared to dsDNA library preparation approaches, ssDNA approaches have yet to be widely adopted in ancient DNA research, mainly because of their higher cost and longer protocol duration compared to double-stranded approaches. For example, the first ssDNA library preparation approach introduced for ancient DNA (Gansauge and Meyer 2013) required CircLigase (Lucigen), a single-stranded DNA ligase that is both expensive and difficult to obtain, and the protocol required 2 days to complete. A revised approach, ssDNA2.0 (Gansauge et al. 2017) (Figure 1B), reduced the expense and protocol duration by replacing the single-stranded ligation step with splinted ligation in which a double-stranded ligation junction is created via hybridization of a double-stranded adapter with a single-stranded degenerate overhang (Kwok et al. 2013). This made it possible to use the widely available and inexpensive T4 DNA ligase rather than CircLigase. While ssDNA2.0 is simpler to implement than the original version, it still requires 4 enzymatic steps and 3 clean-up steps, the latter of which creates opportunities for loss of unique molecules.

We present the Santa Cruz Reaction, or SCR, a fast and inexpensive single-reaction single-stranded DNA library preparation approach that we optimized for ancient DNA (Figure 1C). The SCR is an ancient DNA-specific version of the approach presented by Troll et al. (Troll et al. 2019), in which different enzymatic concentrations, a distinct hybridization strategy, and the use of a dilution series facilitates high-throughput processing of degraded samples. The SCR uses splinted adapters to simultaneously ligate both of the Illumina sequencing adapters in the correct orientation. Because we combine all steps into a single enzymatic reaction, we avoid multiple clean-up steps associated with the loss of unique molecules. To demonstrate the efficacy of the SCR in converting damaged DNA molecules, we use DNA extracted from 5 ancient specimens and prepare libraries using the SCR, BEST, and ssDNA2.0. The SCR converts more molecules than BEST and performs with similar efficiency compared to ssDNA2.0 despite its relative simplicity.

## Materials and Methods

### DNA Extraction

To compare the efficacy of the SCR to other commonly used library preparation approaches in ancient DNA, we prepared DNA extracts from 5 previously characterized ancient bones (4 bison and one horse) that varied in DNA concentration, average fragment length, and deamination frequency (Supplemental Table 1). We powdered each bone using a MM 400 ball mill (Retsch) and performed 4 extractions, each with 100–120 mg of bone powder, from each sample following the silica column-based method described in Dabney et al (Dabney et al. 2013a). We eluted DNA from the column using 50 µL of EBT buffer (10 mm Tris–HCl, 0.05% Tween-20) and pooled the 4 extracts from each sample into a single tube. We then quantified the DNA extraction pools with a Qubit 1X dsDNA HS Assay Kit (Invitrogen, Carlsbad, CA) using 5 µL of DNA extract and a Qubit 4 Fluorometer (Invitrogen). Using these data, we calculated pmols/µL of dsDNA in each pooled extract using an estimated average length of 90 bp for all samples, and pmols/µL of ssDNA or dsDNA ends by multiplying the dsDNA pmol/µL value by 2.

### Library Preparation

We prepared libraries using the SCR, BEST, and ssDNA2.0 library preparation protocols (Figure 1). To assess whether library performance varied by DNA input amounts, we first prepared SCR, BEST, and ssDNA2.0 libraries from the extraction PH158 using 6 different inputs: 1.00 pmols (29.70 ng), 0.50 pmols (14.85 ng), 0.25 pmols (7.43 ng), 0.125 pmols (3.71 ng), 0.063 pmols (1.85 ng), and 0.032 pmols (0.93 ng) of ssDNA or dsDNA ends. Next, we assessed library performance consistency among samples by preparing SCR, BEST, and ssDNA2.0 libraries from each of the 4 remaining DNA extracts using an input of 0.125 pmols (3.71 ng) of DNA. All final pre-amplified libraries were eluted in 50 uL of EBT buffer.

Below, we describe briefly the 3 library preparation protocols. A detailed description of the SCR is provided as supplementary material.

#### Best

The BEST protocol (Figure 1A) is a single-tube double-stranded DNA (dsDNA) library preparation protocol optimized for ancient DNA. We prepared BEST libraries as outlined in Carøe et al. (Carøe et al. 2017) with the modifications described in Mak et al. (Mak et al. 2017), using a 25:1 adapter:template ratio. We used a MinElute column for the final clean-up prior to amplification.

Briefly, BEST libraries are prepared by first performing an end-repair reaction with T4 Polynucleotide Kinase (NEB) and T4 DNA Polymerase (NEB) which blunt-ends the input DNA. Following end-repair, a blunt-end ligation reaction is performed using T4 DNA Ligase (NEB) which facilitates the ligation of the 5′ ends of template molecules to the 3′ ends of blunt-end adapters. Then, an adapter fill-in reaction is performed with Bst 2.0 DNA Polymerase (NEB), which initiates at the ligation junction nick present at the 3′ ends of the template and 5′ ends of the non-ligated adapter strand. Heat inactivation of enzymes occurs between reactions, but a MinElute (Qiagen, Hilden, Germany) column clean-up step is performed following the fill-in reaction.

The BEST protocol flanks the native input DNA molecules with adapters, which will include uracil bases. A uracil-tolerant polymerase must therefore be used during library amplification.

#### ssDNA2.0

SsDNA2.0 (Figure 1B) is a single-stranded library preparation method optimized for damaged and degraded DNA and is the current state-of-the-art ssDNA method for highly degraded samples. We prepared ssDNA2.0 libraries as described in Gansauge et al. (Gansauge et al. 2017) using the TL136 splinter oligo (Supplemental Table 2).

Briefly, ssDNA2.0 libraries are prepared by first dephosphorylating the 5′ and 3′ termini of the input DNA with FastAP (Thermo Scientific, Waltham, MA). The DNA is then heat denatured at 95°C for 1 minute and then rapidly cooled in an ice bath. Once cooled, a biotinylated splinted adapter is ligated to the 3′ end of input DNA using T4 DNA Ligase (Thermo Scientific). The biotinylated adapters, including the ligation products, are then immobilized on C1 beads (Invitrogen), pulled down, and washed. An extension primer is then annealed to the ligated adapter and a second strand is synthesized using the Klenow Fragment (Thermo Scientific), followed by a second C1 bead pull-down and wash step. T4 DNA Ligase (Thermo Scientific) is then used to ligate a double-stranded blunt-end adapter to the 3′ end of the synthesized strand, followed by a third C1 bead pull-down and wash step. Finally, the reactions are heat denatured and the pre-amplified library is collected with the supernatant.

The final adapter-flanked product of an ssDNA2.0 library is the synthesized second strand. Because this will not contain uracil bases, a high fidelity and/or non-uracil tolerant polymerase can be used during library amplification. However, non-standard Illumina oligonucleotide design differences lead to a truncated P5 adapter, which requires the use of a non-standard Illumina sequencing primer.

#### The Santa Cruz Reaction

The Santa Cruz Reaction (SCR; Figure 1C) uses directional splinted ligation of Illumina’s P5 and P7 adapters to convert natively single-stranded DNA and heat denatured double-stranded DNA into Illumina libraries in one enzymatic reaction. Similar to other library preparation protocols, including BEST and NEB Ultra II, the SCR scales the concentration of reaction components to the amount of input DNA to reduce the proportion of adapter-dimers. In the case of the SCR, that includes Extreme Thermostable Single-Stranded Binding Proteins (ET SSB, NEB), which scales with the amount of single-stranded DNA in the reaction. We recommend preparing several splinted adapter and ET SSB dilutions to be used for specific ranges of input DNA (see Supplemental Information).

The SCR begins by combining 20 µL of a DNA extract with 2 µL ET SSB (NEB) at a dilution optimized for the amount of input DNA (see Supplementary Information) to create a sample mixture. The sample mixture is then denatured by heating to 95°C for 3 min, followed by rapid cooling in an ice bath. Next, 1 µL each of P5 and P7 splinted adapters (also at dilutions optimized for the amount of input DNA; see Supplement) are added to the sample mix. Finally, 26 µL of SCR master mix containing 3.75 µL SCR Buffer (666 mm Tris–HCl, 132 mm MgCl_2_), 0.5 µL 100 mm ATP (Thermo Scientific), 0.5 µL 1m DTT (Thermo Scientific), 0.625 µL 2 000 000 U/mL T4 DNA Ligase (NEB), 0.625 µL 10 000 U/mL T4 Polynucleotide Kinase (NEB), and 20 µL 50% PEG 8000 (NEB) is added to the sample mixture, creating a 50 µL reaction. The reaction is pulse-vortexed for 30 s, incubated at 37°C for 45 min, and then cleaned with a MinElute column following the manufacturer’s instructions.

Because the SCR ligates adapters directly to the native input molecules, a uracil tolerant polymerase must be used during library amplification.

The SCR is an ancient DNA-specific version of SRSLY, which was described by Troll et al. (Troll et al. 2019). Several alterations make the SCR more appropriate than SRSLY for converting damaged DNA. For example, the SCR uses DTT and ATP in place of T4 DNA Ligase Buffer (NEB), which appears to better stimulate T4 PNK. Because adapter-dimers are problematic when working with degraded and low-input samples, the SCR also recommends a series of the splinted adapter and ET SSB dilutions for lower DNA input volumes, and implements an asymmetric P5:P7 adapter molar ratio that reduces adapter-dimer formation. Finally, like ssDNA 2.0, the SCR adapter hybridization strategy uses a molar excess of splints to reduce the chance of splintless adapters in the reaction (see Supplementary Information).

### Quantitation, Indexing, and Sequencing

We quantified the amount of library molecules in each library by performing quantitative PCR (qPCR) on a 1:50 dilution of each library using the primers IS7 and IS8 (Gansauge and Meyer 2013), which amplify adapter-ligated templates. We then prepared a 25 µL qPCR for each library using 1 µL of diluted library, 12.5 µL 2X Maxima SYBR Green Master Mix (Thermo Scientific), 10.5 µL H_2_O, 0.5 µL 10 µm IS7 primer, and 0.5 uL 10 µm IS8 primer. Reactions were cycled with the following conditions: 95°C for 10 min, followed by 40 cycles of 95°C for 30 s, 60°C for 30 s, and 72°C for 30 s. Fluorescence was measured at the end of each extension step.

We then performed library amplification and double indexing using the indexing primers described in Kircher et al (Kircher et al. 2012). For each library, we prepared a 100 µL PCR using 2 µL undiluted library, 50 µL Amplitaq Gold 360 Master Mix (Applied Biosystems, Foster City, CA), 1 µL unique 100 µmM i7 indexing primer, 1 µL unique 100 µm i5 indexing primer, and 46 µL H_2_O. We amplified each library with the following cycling conditions: 95°C for 10 min, followed by a library-specific number of cycles of 95°C for 30 s, 60°C for 30 s, and 72°C for 60 s, and a final extension of 72°C for 7 min. We inferred the optimal cycle number for each library from the qPCR results (Supplemental Table 3). We used each library’s CT value, rounded to the nearest cycle, to determine the optimal number of cycles for indexing PCR.

We purified the amplified libraries using 120 µL (1.2×) of a SPRI bead mixture, which we prepared according to and performed as described in Rohland and Reich (Rohland and Reich 2012). We quantified the purified libraries with the Qubit 1X dsDNA HS Assay Kit and Qubit 4, and visualized the products using a D1000 ScreenTape (Agilent, Santa Clara, CA) and Tapestation 2200 (Agilent).

We sequenced each library at the University of California, Santa Cruz Ancient and Degraded DNA Processing Center using 150 cycle mid output kits on an Illumina NextSeq 550. Because they needed a non-standard primer, we sequenced libraries prepared with ssDNA2.0 on separate runs with a complete replacement of Illumina’s read 1 sequencing primer with the oligo CL72, as described in Gansauge et al. (Gansauge and Meyer 2013). We performed base calling using Illumina’s bcl2fastq2 software.

### Data Analysis

To compare the performance of the 3 library preparation protocols, we downsampled fastq files from each library to the number of reads generated from the least deeply sequenced library per library preparation approach. We merged reads that overlapped by at least 15-bases, trimmed adapters, and removed reads that were under 30 bp long using SeqPrep (https://github.com/jstjohn/SeqPrep). We then mapped merged and unmerged reads separately using Burrows–Wheeler Aligner (BWA) (Li and Durbin 2009) v0.7.12 aln algorithm with seed disabled to either the equCab2 (Wade et al. 2009) or bison_umd1.0 (GCA_000754665.1) reference genomes, depending on whether the sample was a horse or a bison. We collapsed PCR duplicates and generated mapping summary statistics using SAMtools (Li et al. 2009). We calculated read lengths directly from the merged reads and, for unmerged reads, inferred total lengths based on mapping coordinates. We computed cytosine deamination frequencies using mapDamage2.0 (Jónsson et al. 2013).

## Results

### Library Conversion Efficiency

We used qPCR and the proportion of unique mapped reads to estimate library conversion efficiency. As the number of amplifiable molecules in a library increases, fewer cycles are necessary for the library to reach a detection threshold during qPCR. The cycle threshold (CT value) is the cycle number at which a library reaches this detection threshold. A library that is detected one CT value sooner than another has approximately twice the number of amplifiable starting molecules than the later-detected library.

**Figure 2.**
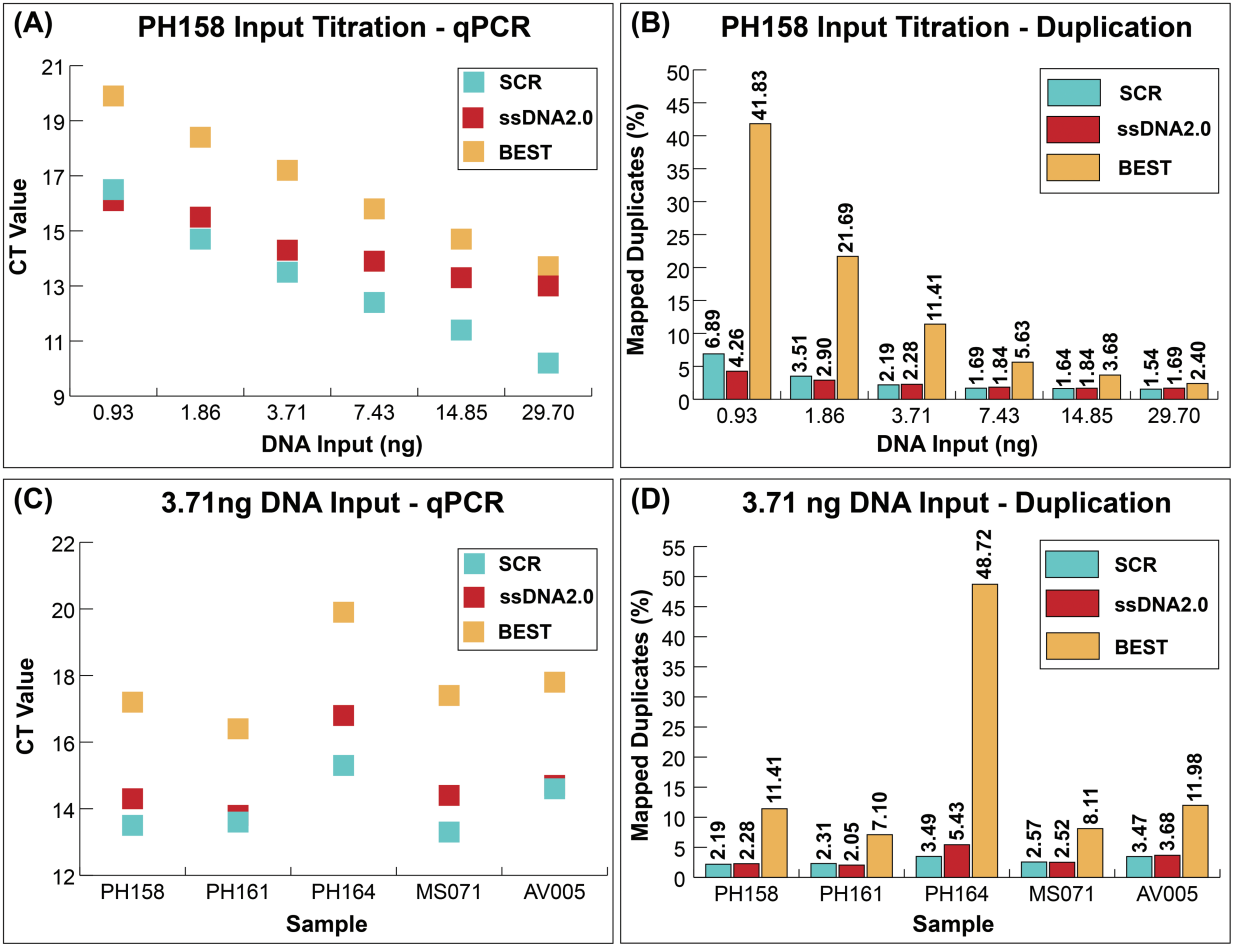
Library preparation complexity comparison. (**A**) Quantitative PCR CT values for libraries prepared from sample PH158 using a titration of 6 DNA inputs ranging from 0.93 ng to 29.70 ng. Lower CT values indicate more starting library molecules in the reaction (**B**) Proportion of mapped reads that are duplicates prepared from sample PH158 at the different titrations of DNA input. (**C**) Quantitative PCR CT values for libraries prepared from 5 ancient DNA extracts using a static DNA input of 3.71 ng. (**D**) Proportion of duplicated reads from libraries prepared from these 5 ancient DNA extracts using the static 3.71 ng DNA input.

When comparing CT values for libraries from PH158 prepared using 6 different DNA input volumes, both ssDNA library preparation methods converted more molecules compared to the double-stranded approach (Figure 2A). The ssDNA library approaches performed similarly, with SCR recovering more molecules among the higher input libraries and ssDNA2.0 recovering more molecules among the lower input libraries. At the highest DNA input, 29.70 ng or 1.00 pmol ssDNA, the SCR library reached the detection threshold 2.8 cycles earlier than ssDNA2.0, suggesting that 7.0X more DNA was converted. At increasingly lower DNA inputs, the CT value difference between SCR and ssDNA2.0 decreased. At the lowest DNA input, 0.93 ng or 0.037 pmol ssDNA, the ssDNA2.0 library reached the detection threshold 0.4 cycles earlier than SCR, suggesting that ssDNA2.0 converted 1.3X more DNA than the SCR.

Because qPCR cannot discriminate between adapter-dimers and adapter-flanked molecules, we next compared, as a measure of library complexity, the proportion of reads that mapped to the reference genome that are duplicates (1 – [# unique mapped reads] / [# total mapped reads]). This allows us to distinguish libraries that convert more unique molecules as those that have a lower proportion of mapped duplicated reads. After down sampling each library to equal numbers of reads, we observed a trend similar to that from qPCR in which the SCR and ssDNA2.0 libraries contain a lower proportion of mapped duplicates (and therefore a higher proportion of unique reads) compared to the BEST libraries (Figure 2B). While the 2 ssDNA library preparation approaches performed similarly to each other, we observed some differences with DNA input. The SCR libraries contained a lower proportion of mapped duplicates compared to the ssDNA2.0 libraries at the higher DNA inputs, while ssDNA2.0 libraries contained a lower proportion of mapped duplicates compared to SCR at low (1.86 ng and 0.93 ng) inputs. Because the 2 ssDNA library preparation protocols have similar conversion efficiency at higher DNA inputs, we selected a higher DNA input for the remaining library comparisons.

When comparing CT values given a static 3.71 ng DNA input across the 5 DNA extracts, we observed similar trends between library preparation approaches to those reported above. The 2 single-stranded approaches reach the detection threshold before BEST (Figure 2C) across all 5 extracts. The SCR libraries reached the detection threshold between 0.1 and 1.5 cycles before the ssDNA2.0 libraries, suggesting that SCR converted 1.1X - 2.8X more molecules at this input volume.

After down sampling each library to an equal number of reads, we observed the 2 single-stranded approaches contained a lower proportion of mapped duplicate reads than the double-stranded approach for all 5 extracts (Figure 2D). The single-stranded approach that produced the lowest proportion of duplicates varied by extract, suggesting that the 2 ssDNA approaches are similarly efficient at this DNA input (3.71 ng). While the SCR produced libraries with a lower proportion of mapped duplicates in 3 of 5 extracts, qPCR suggested that SCR converts more DNA to library compared to ssDNA2.0 across all 5 extracts. The discrepancy between the qPCR and sequencing results is most likely due to the higher proportion of adapter-dimers in the SCR libraries compared to the ssDNA2.0 libraries

### Endogenous Content, Average Fragment Length, and Terminal Deamination Frequency

**Figure 3.**
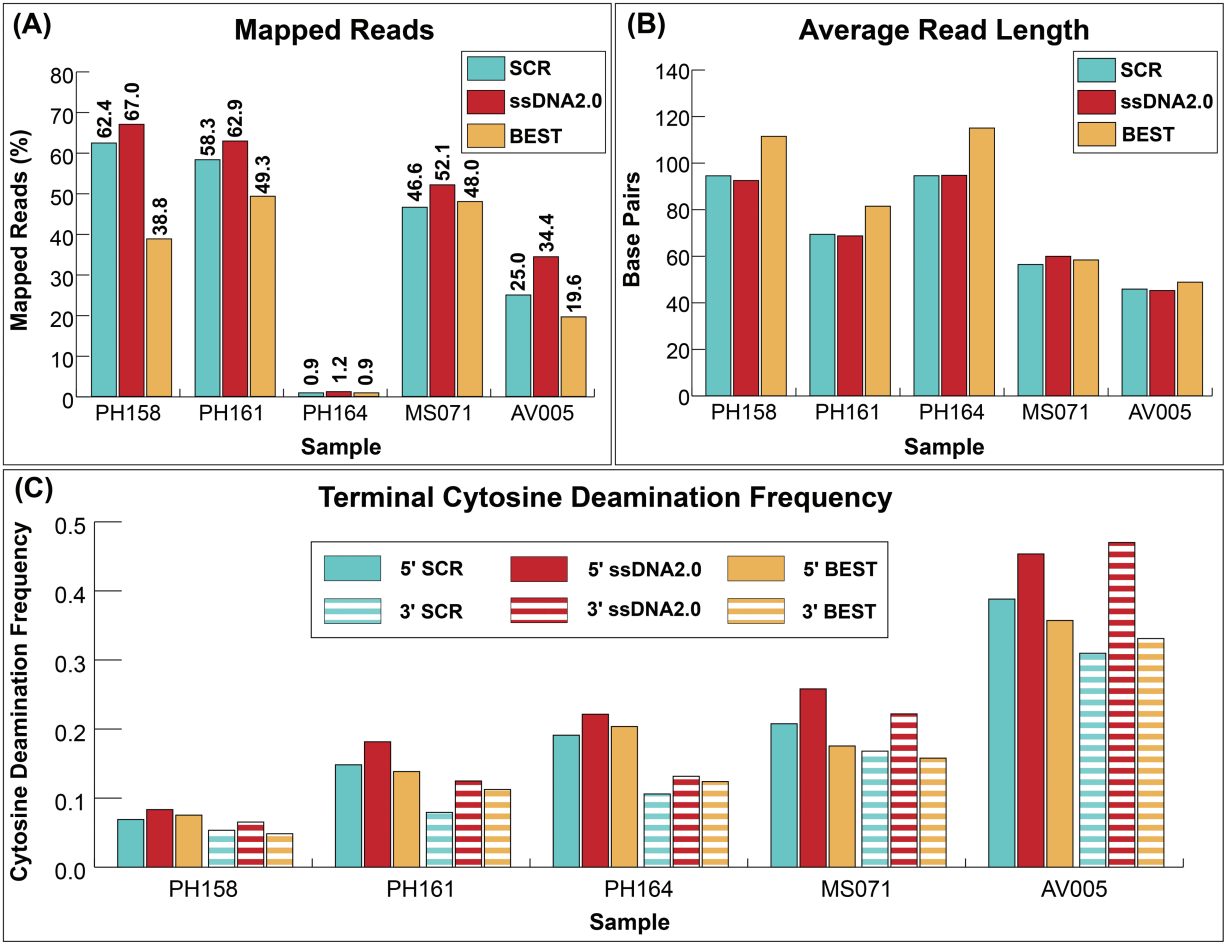
Sequencing statistics for libraries prepared from 5 samples using 3.71 ng of input DNA. (**A**) The percentage of reads mapped to the reference genome. (**B**) The average length of all mapped reads. (**C**) The terminal 5′ and 3′ cytosine deamination frequencies of the mapped reads.

Next, we compared the endogenous DNA content, the proportion of DNA that mapped to the relevant reference genome, in each library. The 2 ssDNA library approaches recovered either more or a similar proportion of endogenous DNA compared to the dsDNA library approach for all 5 extracts (Figure 3A). SsDNA2.0 recovered the highest proportion of endogenous DNA for all extracts, and SCR recovered between 72.8% and 93.1% of that recovered by ssDNA2.0.

The 2 ssDNA methods produced libraries with similar average fragment lengths and, for most samples, shorter average fragment lengths compared to BEST. The average fragment length difference between the SCR and ssDNA2.0 libraries ranged from 0.18 bp to 3.52 bp (Figure 3B), and both approaches resulted in a similar fragment length distribution (Figure S2). The BEST libraries had a noticeably higher average fragment length compared to SCR when the extracted DNA was less fragmented. However, BEST libraries produced from the 2 most heavily fragmented samples, AV005 and MS071, had a similar average fragment length compared to the SCR.

Libraries prepared with ssDNA2.0 have a consistently higher frequency of terminal deamination on both the 5′ and 3′ ends compared to the SCR and BEST libraries (Figure 3C). We also observed a terminal deamination asymmetry in nearly all libraries in which the 5′ end contains a higher rate of deamination compared to the 3′ end. Libraries prepared with the SCR exhibit the highest deamination asymmetry in all but AV005, which also has the shortest average fragment length and is likely to be the most degraded of the 5 extracts.

## Discussion

Ancient DNA research often involves screening tens to hundreds of samples for preserved DNA at the outset of a research project. For most samples, it is impossible to know whether the sample will be sufficiently well-preserved to generate genome-scale data without extracting DNA, preparing that DNA extract into a sequenceable library, and sequencing that library. Although single-stranded library preparation approaches are understood to be more efficient in converting ancient DNA molecules into sequenceable form than are double-stranded library preparation approaches (Bennett et al. 2014; Wales et al. 2015; Gansauge et al. 2017), ssDNA library preparation approaches have yet to be widely adopted in ancient DNA research labs because they are more expensive and take considerably longer to complete compared to dsDNA library preparation methods. The Santa Cruz Reaction solves this problem by converting ancient DNA molecules into sequencing libraries with an efficiency that is comparable to the current state-of-the-art ssDNA approach, ssDNA2.0. Compared to ssDNA2.0, however, SCR reduces ssDNA library preparation to a single cost-effective enzymatic step that focuses on the primary goal of fragmented DNA library preparation, adapter ligation. Reducing the number of protocol steps reduces the duration of the pre-amplification protocol by 2.5 times compared to ssDNA2.0. We also note that time to completion can be further reduced by replacing the column-based cleanup used here with a magnetic bead clean-up.

We highlight several challenges associated with the SCR protocol. First, because we add 4 oligonucleotides to a single reaction, including a phosphorylated adapter, adapter-dimers form more readily compared to ssDNA2.0 and BEST (Table S3). To reduce the proportion of adapter-dimers in the final library, titration of adapters to input DNA is beneficial. We have developed an adapter dilution series, where 5 adapter concentrations are used across specific ranges of DNA inputs. Second, batches of synthesized splint oligonucleotides often include synthesis artifacts that render DNA fragments capable of ligation at ends that should be blocked for ligation (Figure S1). While this issue was noted previously along with an oligonucleotide purification strategy (Gansauge et al. 2017, 2020), we were not able to successfully adopt an artifact removal scheme from our synthesized splint oligonucleotides. Instead, we implemented several oligonucleotide usage optimizations such as a quality control procedure (See Supplemental Protocol), which allows users to identify poor quality splint batches prior to use. Furthermore, we have optimized the adapter:splint hybridization ratio and use asymmetrical P5:P7 adapter concentrations in the reaction, this reduces the most detrimentally volatile reaction component, the P7 splint, without hindering library preparation performance. Future oligo design improvements may allow for further streamlining of SCR reagent preparation and usage.

In agreement with previous studies (Bennett et al. 2014; Wales et al. 2015; Gansauge et al. 2017), we found the ssDNA library preparation methods convert more molecules to library compared to the dsDNA method across all DNA input amounts and the 5 ancient DNA extracts used here. The differences in conversion efficiencies between the 2 single-stranded methods are more nuanced. Both qPCR and sequencing results from the input titration experiment suggest the SCR and ssDNA2.0 outperform each other at opposite ends of the DNA input spectrum, with ssDNA2.0 outperforming SCR at the lowest input amounts. The SCR’s lower library conversion efficiency at the lowest input amounts is likely due to too low P5 and P7 adapter concentrations in the reaction at lowest adapter dilution tier. The scaling of the adapters with the amount of input DNA is a challenge and, at present, a necessity. We note that the higher proportion of adapter-dimers in the SCR libraries may lead to an inflation of qPCR-based estimates of converted DNA. This could be explored further by sequencing each library to exhaustion, however the high complexity of most ssDNA libraries made this impractical.

Interestingly, the 2 ssDNA library methods appear to convert slightly different populations of input molecules to the final library. In particular, ssDNA2.0 libraries consistently have higher terminal deamination frequencies compared to the SCR libraries (Figure S3). This may indicate that ssDNA2.0 is better able to convert and retain molecules containing a terminal uracil, which may partly explain the higher endogenous content of ssDNA2.0 libraries compared to SCR. The differences in terminal deamination frequency may be driven by ligation scheme, in which the splinted adapter targeting the 3′ end during the SCR is in approximately 6X molar excess compared to the input DNA and in approximately 80X molar excess in ssDNA2.0. Splint species that are highly reactive to uracil containing termini, such as those with an adenine at the ligation junction, may become limiting when splint molar excess is low. This hypothesis could be tested by altering the base composition of the splint bases near the ligation junction to contain higher adenine content. We also observed that the mapped reads from the SCR libraries have an average GC content that more closely reflects the reference genome compared to libraries prepared with ssDNA2.0 (Figure S4). This may be caused by polymerase GC bias during the second strand synthesis of ssDNA2.0.

We present a protocol for fast and simple DNA library preparation that can recover degraded molecules preserved in both single-stranded and double-stranded forms. Although ssDNA2.0 outperforms the SCR at the lowest input volumes and may be more appropriate for the most degraded samples, the SCR performs as well as ssDNA2.0 across a wide range of input volumes and is an appropriate and more efficient replacement for commonly used dsDNA library preparation approaches.

## Supplementary Material

esab012_suppl_Supplementary_Material

## Data Availability

Fastq files are available online at Dryad, DOI: https://doi.org/10.7291/D1M388.
