# Supplementary material for: A Fast and Efficient Single-stranded Genomic Library Preparation Method Optimized for Ancient DNA
Source: J Hered. 2021 Mar 25;112(3):241–9. doi: 10.1093/jhered/esab012 (PMC8141684; doi:10.1093/jhered/esab012)
Supplement: esab012_suppl_Supplementary_Material [file esab012_suppl_supplementary_material.pdf]

Supplemental Material

The Santa Cruz Reaction Protocol

S1. Oligonucleotides (IDT Format, 5' → 3')

Note: All oligonucleotides are ordered with HPLC purification conducted by IDT.
Note: We recommended ordering two separate batches of each splint oligonucleotide. See oligonucleotide quality control recommendations in section 11 of this protocol.

scr_P5_adapter: /5AmMC12/ACACTCTTTCCCTACACGACGCTCTTCCGATCT
scr_P7_adapter: /5Phos/AGATCGGAAGAGCACACGTCTGAACTCCAGTCAC/3AmMO/
scr_P5_splint: /5AmMC6/NNNNNNNAGATCGGAAGAGCGTCGTGTAGGGAAAGAGTGT/3AmMO/
scr_P7_splint: /5AmMC12/GTGACTGGAGTTCAGACGTGTGCTCTTCCGATCTNNNNNNN/3AmMO/

S2. Reaction Reagents

Reagent	Provider	Cat #
T4 DNA Ligase (2,000,000 U/mL)	NEB	M0202M
T4 PNK (10,000 U/mL)	NEB	M0201L
ET SSB (500 ng/µL)	NEB	M2401S
ATP (100 mM)	Thermo Scientific	R0441
DTT (1 M)	Thermo Scientific	P2325
T4 RNA Ligase Buffer	NEB	B0216L
PEG 8000 (50%)	NEB	B0216L
Tris-HCl pH 8.0 (1M)	Invitrogen	15568025
EDTA pH 8.0 (0.5M)	Invitrogen	15575020
Tween-20 (10%)	Teknova	T0710
NaCl (5M)	Sigma-Aldrich	S5150-1L
Glycerol (50%)	Invitrogen	15514011
$MgCl_2$ (1M)	Invitrogen	AM9530G

S3. Buffer Preparation

- **TE Buffer (Store at RT):** 10 mM Tris-HCl (pH 8.0), 1 mM EDTA (pH 8.0)
- **EBT Buffer (Store at RT):** 10 mM Tris-HCl (pH 8.0), 0.05% Tween-20
- **Adapter Dilution Buffer (Store at -20˚C):** 1X T4 RNA Ligase Buffer, 0.05% Tween-20
- **SSB Dilution Buffer (Store at -20˚C):** 20 mM Tris-HCl (pH 8.0), 20 mM NaCl, 0.5 mM DTT, 0.1 mM EDTA (pH 8.0), 50% Glycerol
- **SCR Buffer (Store at -20 °C):** 666 mM Tris-HCl, 132 mM $MgCl_2$

S4. Adapter-Splint Hybridizations (Store hybridized stocks at -20 °C)

1. Resuspend all oligonucleotides to 100 µM using TE buffer.

2. Add the following components to a 0.2 mL tube labeled P5:
   - **H_2O:** 30.6 µL
   - **10X T4 RNA Ligase Buffer:** 5 µL
   - **100 µM scr_P5_adapter:** 6 µL
   - **100 µM scr_P5_splint:** 8.4 µL

3. Add the following components to a 0.2 mL tube labeled P7:
   - **H_2O:** 30.6 µL
   - **10X T4 RNA Ligase Buffer:** 5 µL
   - **100 µM scr_P7_adapter:** 6 µL
   - **100 µM scr_P7_splint:** 8.4 µL

4. In a thermocycler with heated lid (105 °C), hybridize the adapters and splints by incubating at 95 °C for 1 minute before ramping down to 10 °C at 0.1 °C per second.

5. Store hybridized P5 (12 µM) and P7 (12 µM) adapter stock solutions at -20 °C.

S5. Adapter-Splint Dilutions (Store dilutions at -20 °C)

Note: Freeze-thaw adapter dilutions no more than five times.

1. Prepare working **P5** adapter solutions by diluting hybridized 12 µM stock solutions to the following molarities. Dilute with Adapter Dilution Buffer.
   - **6.0 µM**
   - **3.0 µM**
   - **1.5 µM**
   - **0.75 µM**

2. Prepare working **P7** adapter solutions by diluting hybridized 12 µM stock solutions to the following molarities. Dilute with Adapter Dilution Buffer.
   - **6.0 µM**
   - **3.0 µM**
   - **1.5 µM**
   - **0.75 µM**
   - **0.375 µM**

S6. ET SSB Dilutions (Store dilutions at -20 °C)

1. Prepare the following **ET SSB** dilutions from **500 ng/µL** stock solution using SSB Dilution Buffer:
   - **328 ng/µL**
   - **164 ng/µL**
   - **82 ng/µL**
   - **41 ng/µL**
   - **20.5 ng/µL**

S7. Reaction Mix Preparation (Store reaction mix at -20 °C)

Notes:

- Freeze-thaw the Reaction Mix no more than five times.
- Warm PEG 8000 to 50 °C before pipetting into the Reaction Mix tube as the first component.
- Equilibrate PEG 8000 to RT before adding remaining reagents.
- Thoroughly mixing the Reaction Mix is essential, vortexing is recommended.

Reagent	Stock Conc.	1 RXN (µL)	Final Conc.	20 RXNs (µL)
PEG 8000	50%	20	20%	400
SCR Buffer	13.3X	3.75	1X	75
DTT	1M	0.5	10mM	10

ATP	100mM	0.5	1mM	10
T4 PNK	10,000 U/mL	0.625	0.125 U/mL	12.5
T4 DNA Ligase	2,000,000 U/mL	0.625	25 U/mL	12.5

S8. Reaction Adapter-Splint and SSB Input Tiers

picomoles ssDNA	dsDNA* (ng)	P5 (μM)	P7 (μM)	SSB (ng/μL)
1.0 - 2.5	29 - 75	12	6	328
0.5 - 0.99	15 - 29	6	3	164
0.25 - 0.49	7 - 15	3	1.5	82
0.12 - 0.24	3 – 7	1.5	0.75	41
< 0.12	< 3	0.75	0.375	20.5

***Approximate degraded dsDNA input. The average size used to calculate the number of picomoles is deliberately overestimated due to the difficulties of accurately visualizing highly degraded samples.**

S9. The Santa Cruz Reaction (SCR) Workflow

Note: The SCR uses a tiered adapter and SSB system based on the DNA input into the reaction. Use the table in **Section 8** to select the correct dilution set for each DNA extract.

Critical Note: Thoroughly mixing the reaction in steps seven and eight is essential to consistently achieve high ligation efficiency. Inadequately mixing the reaction is the most common failure mode. Vortexing is recommended.

1. Gather the following reagents and equipment:
   - Thaw and equilibrate the Reaction Mix Preparation and appropriate SSB dilution to room temperature before pipetting.
   - Thaw the appropriate P5 and P7 adapter-splint dilutions and place them on ice.
   - Prepare an ice bath.

2. Prepare the Sample Mix by combining the following in a 0.2 mL PCR tube, 8-tube strip, or 96-well plate:
   - **DNA Extract:** 20 μL (if less than 20 μL, fill to 20 μL with buffer EBT)
   - **Appropriate SSB Dilution:** 2 μL

3. Pulse-vortex the Sample Mix five times at maximum speed prior to briefly spinning down in a mini centrifuge.

4. Incubate the Sample Mix at 95 °C for 3 minutes before immediately placing the Sample Mix into an ice bath.

5. Allow the Sample Mix to cool on ice for 2 minutes, spin down briefly in a mini centrifuge, and return the Sample Mix to the ice bath.

6. While on ice, add the following to the cooled Sample Mix:
   - **Appropriate P5 Adapter Dilution:** 1 μL
   - **Appropriate P7 Adapter Dilution:** 1 μL

- **Reaction Mix Preparation:** 26 µL

7. Pulse-vortex the reaction five times at maximum speed and then spin down briefly in a mini centrifuge.

8. Repeat step 7 two more times.

9. Incubate the reaction at 37 °C for 45 minutes.

10. Proceed directly to reaction clean-up:
    - To retain the shortest molecules, use a MinElute PCR Purification Kit (Qiagen Catalog No. 28004) following the manufacturer's instructions. Elute in 50 µL buffer EBT.

11. The purified reaction products are ready for indexing and amplification by PCR.
    - **Note:** Use of a uracil-tolerant polymerase is required when working with degraded DNA as a starting material. Examples include: AmpliTaq Gold (ThermoFisher), Pfu Turbo cx (Agilent), KAPA Uracil+ (Roche), and Q5U (NEB).

12. A 1.2X SPRI purification is the recommended post-PCR purification strategy.

S10. Oligonucleotide Quality Control - Workflow

Note: The oligonucleotides for the Santa Cruz Reaction are designed with blocking modifications to limit undesirable ligation products. However, the splint oligonucleotides may arrive with one or more subspecies containing unblocked termini. Currently, a consistent purification strategy to eliminate poor quality splint species does not exist. However, this section presents a ligation based method to identify poor quality splint batches.

Briefly, splint oligonucleotides are spiked into Santa Cruz Reactions. Ligatable and amplifiable species within the splint spike will convert to library molecules, which will be identifiable during post amplification visualization. A splint batch with a high proportion of ligatable and amplifiable species should not be used for the library preparation of ancient samples (see section 11 for trace interpretation guidance).

Each new batch of P5 and P7 splint should undergo the quality control scheme below.

1. Follow the adapter hybridization, reagent preparation, and dilution guidelines in Sections 3-7.

2. Prepare a number of Santa Cruz Reactions equal to the number of freshly synthesized splint batches requiring quality control plus one blank
    - **Example**: 1 P5 Splint + 1 P7 Splint + 1 Blank = 3 reactions.

3. In a 0.2 mL PCR 8-tube strip add the following to wells 1-3.
    - **Well 1:** 1 pmol P5 splint and fill to 20 µL (5 µL of 0.2 µM P5 splint + 15 µL EBT)
    - **Well 2:** 1 pmol P7 splint and fill to 20 µL (5 µL of 0.2 µM P7 splint + 15 µL EBT)
    - **Well 3:** 20 µL EBT

4. Add 2 µL of the 20.5 ng/µL SSB dilution to each well.

5. Follow steps 3-5 of the SCR workflow in section eight to mix, heat denature, and chill the reactions.

6. Add the following components to each chilled reaction:
- **0.75 µM P5 Hybridized Adapter Dilution:** 1 µL
- **0.375 µM P7 Hybridized Adapter Dilution:** 1 µL
- **Reaction Mix:** 26 µL

7. Follow steps 7-9 of the SCR workflow in section eight to properly mix and incubate the reactions.

8. Following incubation at 37 °C, clean each reaction using the MinElute PCR Purification Kit according to the manufacturer's instructions. Elute in 50 µL buffer EBT.

9. Amplify and index the entire eluate from step 8 for 18 cycles using the PCR scheme of your choice.

10. Clean each amplified library with the MinElute PCR Purification Kit according to the manufacturer's instructions.

11. Visualize each library, including the negative control, on a Fragment Analyzer, TapeStation, or BioAnalyzer automated electrophoresis system.

S12. Oligonucleotide Quality Control - Interpretation

Note: Clean splint batch libraries contain adapter-dimers and a small proportion of secondary oligonucleotide artifacts, which should resemble panel A and B in Figure S1.
Note: Poorly synthesized splint batch libraries contain adapter-dimers and an unpredictable number of secondary oligonucleotide artifacts, which make-up a large portion of the library. Panel C and D in Figure S1 are examples or poorly synthesized splint batches, which should be discarded to avoid artifacts from contaminating the final libraries.

1. **Compare the negative control trace to panels A and B in Figure S1.**
    - The adapter-dimer peak, around 130-140 bp, should dominate the trace but a small proportion of secondary peaks starting directly to the right of the adapter dimer and extending to 280bp may also be present.
    - The splint oligonucleotides are ready for use if the negative control is comparable to panels A and B in Figure S1.
    - See Step 2 if peaks to the right of the adapter-dimer peak dominate the negative control trace.

2. **Compare the P5 and P7 splint input traces to the negative control trace.**
    - Between the P5 splint trace and negative control trace look for a change in the proportions of each peak. Do the same between the P7 splint trace and the negative control trace.
    - An observed decrease in the total proportion of the adapter-dimer peak (increase in secondary peaks) in one of the splint traces indicates the presence of undesirable oligonucleotide contaminants.
    - Quarantine or discard splint batches that have produced significant secondary oligonucleotide ligation artifacts.

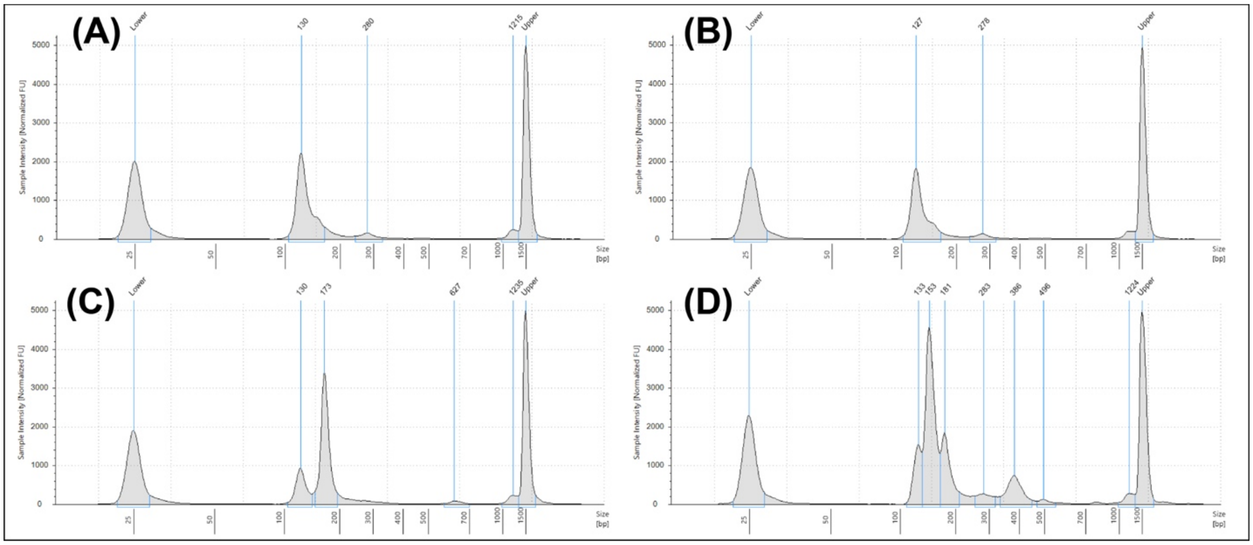

Figure S1. Four P5 splint oligonucleotide spike-in reaction TapeStation traces. (A) and (B) are P5 splint batches that contain acceptable levels of oligonucleotide secondary artifact species. (C) and (D) are P5 splint batches that have high levels of oligonucleotide secondary artifact species and should be quarantined from normal library use or discarded.

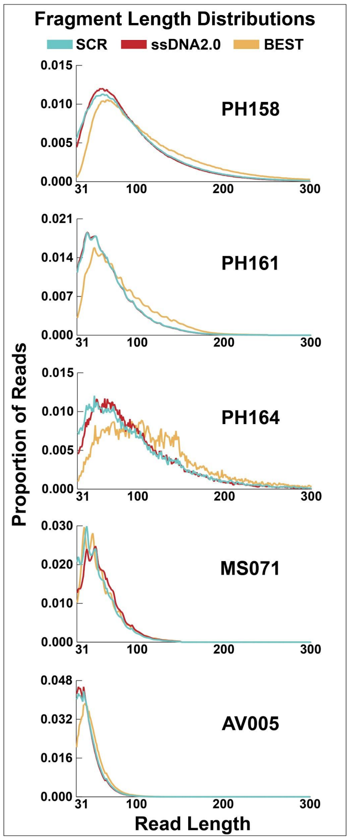

Figure S2. Length distribution of reads mapped to the reference genome.

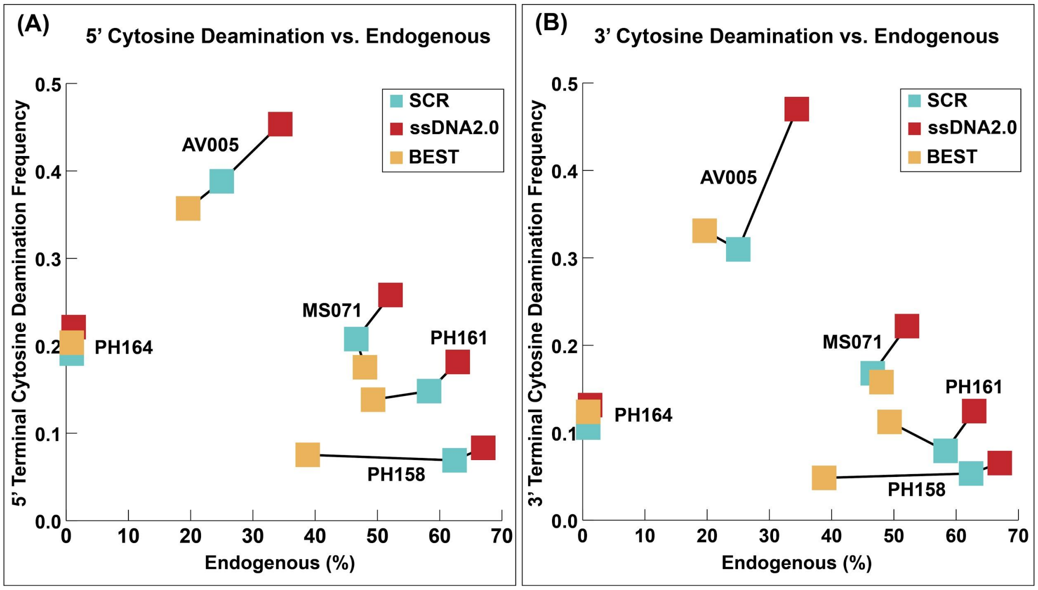

Figure S3. Scatter plots of **(A)** 5’ terminal deamination frequency vs. the percentage of endogenous reads in the library and **(B)** 3’ terminal deamination frequency vs. the percentage of endogenous reads library.

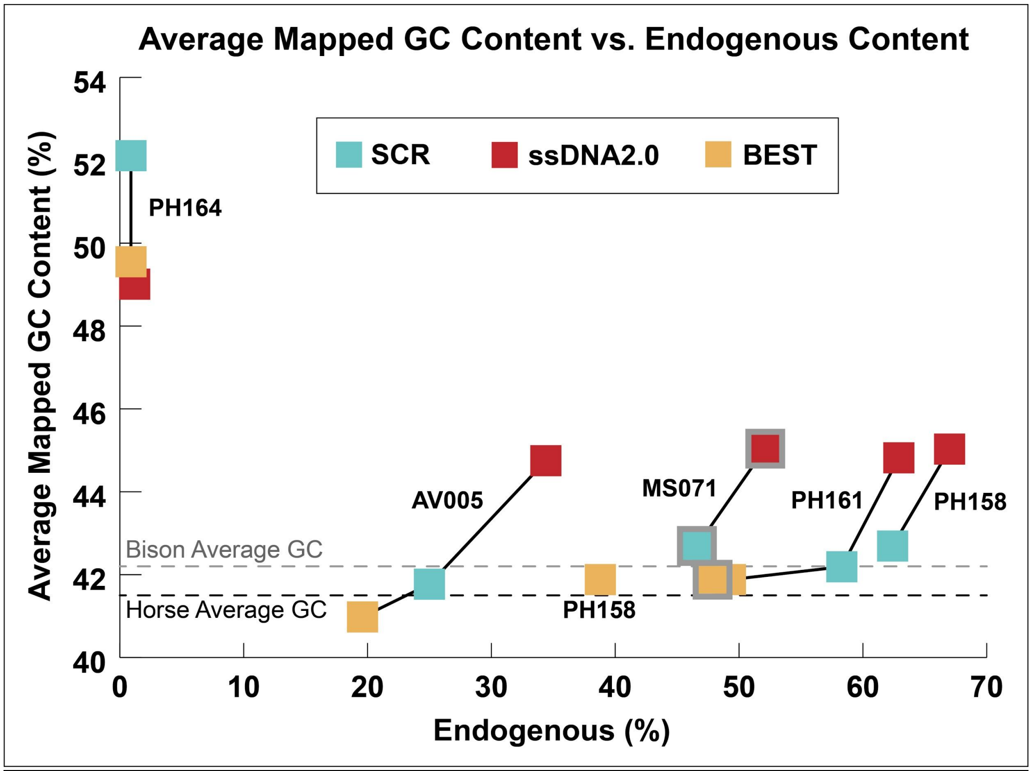

Figure S4. Scatter plot of the average GC content of mapped reads vs. the percentage of endogenous reads in the library.

UCSC ID	Museum ID	Organism	Age (C14 date)	Location	Bone Powder Per Extract (mg)	Extracts Performed	Pool Conc. (ng/µL)
PH158	F-2431	Horse	40,680	Yakutiya, Russia	100	4	3.77
PH161	F-2530	Horse	Not Dated	Yakutiya, Russia	120	4	1.26
PH164	F-2538	Horse	Not Dated	Yakutiya, Russia	120	4	4.07
MS071	YG 303.666	Bison	16,390	Yukon Territory, Canada	120	4	0.52
AV005	YG412.17	Horse	Not Dated	Yukon Territory, Canada	120	4	0.60

Table S1. Overview of the ancient samples used for DNA extraction and library preparation.

Method	Oligo Name	Provider	Purification	Sequence 5'->3'
SCR	scr_p5_adapter	IDT	HPLC	/5AmMC12/ACACTCTTTCCCTACACGACGCTCTTCCGATCT
	scr_p5_splint	IDT	HPLC	/5AmMC6/NNNNNNNAGATCGGAAGAGCGTCGTGTAGGGAAAGAGTGT/3AmMO/
	scr_p7_adapter	IDT	HPLC	/5Phos/AGATCGGAAGAGCACACGTCTGAACTCCAGTCAC/3AmMO/
	scr_p7_splint	IDT	HPLC	/5AmMC12/GTGACTGGAGTTCAGACGTGTGCTCTTCCGATCTNNNNNNN/3AmMO/
ssDNA2.0	CL78	Sigma	Ion-exchange HPLC (Dual)	Pho-AGATCGGAAG[C3Spacer]10-TEG-biotin
	TL136	Sigma	RP-HPLC	SpacerC12-AA[SpacerC12]CTTCCGATCTNNNNNNNN-AmC6
	CL53	Sigma	RP-HPLC	CGACGCTCTTC-ddC
	CL73	Sigma	RP-HPLC	Pho-GGAAGAGCGTCGTGTAGGGAAAGAG*T*G*T*A
	CL130	Sigma	RP-HPLC	GTGACTGGAGTTCAGACGTGTGCTCTTCC*GA*TC*T
BEST	IS1_BEDC3	IDT	HPLC	A*C*A*C*TCTTTCCCTACACGACGCTCTTCCG*A*T*C*T
	IS2_BEDC3	IDT	HPLC	G*T*G*A*CTGGAGTTCAGACGTGTGCTCTTCCG*A*T*C*T
	IS3_BEDC3	IDT	HPLC	A*G*A*T*CGGAA*G*A*G*C/3SpC3/
qPCR	IS7	IDT	Desalted	ACACTCTTTCCCTACACGAC
	IS8	IDT	Desalted	GTGACTGGAGTTCAGACGTGT

Table S2. Information for oligonucleotides used during library preparation.

Method	Lib ID	Sample ID	Organism	Lib Input (ng)	Lib Input (pmol)	qPCR (CT)	Pre-Amp Vol (µL)	iPCR Input (µL)	Cycles Amplified	Ref Genome	Raw Reads	Adapter-Dimers (%)	Sequences >30bp (%)	Mapped Sequences Over 30bp (%)	Mapped Unique Sequences (%)	Average Size of Mapped Sequences (bp)	C-to-T Sub Freq 5' end (%)	C-to-T Sub Freq 3' end (%)
SCR	JKFC1	PH158	Horse	29.70	1.000	10.2	50	2	10	equCab2	11,000,000	1.82	93.07	61.65	98.46	94.90	7.05	6.04
	JKFC2	PH158	Horse	14.85	0.500	11.4	50	2	11	equCab2	11,000,000	1.69	93.18	61.28	98.36	95.70	7.06	5.96
	JKFC3	PH158	Horse	7.43	0.250	12.4	50	2	12	equCab2	11,000,000	1.32	93.96	61.06	98.31	96.44	7.02	5.88
	JKFC4	PH158	Horse	3.71	0.125	13.5	50	2	14	equCab2	11,000,000	1.18	94.19	62.39	97.81	94.48	6.90	5.34
	JKFC5	PH158	Horse	1.86	0.063	14.7	50	2	15	equCab2	11,000,000	1.11	94.59	63.95	96.49	94.49	6.24	4.15
	JKFC6	PH158	Horse	0.93	0.031	16.5	50	2	16	equCab2	11,000,000	2.25	92.13	62.44	93.11	93.13	5.79	3.68
	JKFC8	PH161	Horse	3.71	0.125	13.6	50	2	14	equCab2	13,000,000	0.82	92.25	58.33	97.69	69.35	14.82	7.93
	JKFC10	PH164	Horse	3.71	0.125	15.3	50	2	15	equCab2	7,200,000	7.31	83.50	0.92	96.51	94.50	19.11	10.60
	JKFC12	MS071	Bison	3.71	0.125	13.3	50	2	13	bison_umd1.0	12,000,000	0.81	90.82	46.57	97.43	56.38	20.77	16.80
	JKFC14	AV005	Horse	3.71	0.125	14.6	50	2	15	equCab2	12,000,000	1.82	86.68	25.04	96.53	45.82	38.81	30.97
ssDNA2.0	JKFC57	PH158	Horse	29.70	1.000	13.0	50	2	13	equCab2	11,000,000	0.14	98.02	69.78	98.31	101.22	8.93	7.00
	JKFC58	PH158	Horse	14.85	0.500	13.3	50	2	13	equCab2	11,000,000	0.18	97.50	69.02	98.32	98.14	8.67	6.98
	JKFC59	PH158	Horse	7.43	0.250	13.9	50	2	14	equCab2	11,000,000	0.19	96.76	68.01	98.16	92.95	8.58	6.89
	JKFC60	PH158	Horse	3.71	0.125	14.3	50	2	14	equCab2	11,000,000	0.19	95.52	67.02	97.72	92.46	8.33	6.53
	JKFC61	PH158	Horse	1.86	0.063	15.5	50	2	15	equCab2	11,000,000	0.21	92.64	66.54	97.10	89.12	8.35	6.58
	JKFC62	PH158	Horse	0.93	0.031	16.1	50	2	16	equCab2	11,000,000	0.25	89.99	66.14	95.74	90.46	8.32	6.49
	JKFC64	PH161	Horse	3.71	0.125	13.8	50	2	14	equCab2	13,000,000	0.16	92.91	62.90	97.95	68.69	18.15	12.48
	JKFC66	PH164	Horse	3.71	0.125	16.8	50	2	17	equCab2	7,200,000	0.42	89.37	1.18	94.57	94.68	22.14	13.16
	JKFC68	MS071	Bison	3.71	0.125	14.4	50	2	14	bison_umd1.0	12,000,000	0.21	93.70	52.09	97.48	59.90	25.81	22.19
	JKFC70	AV005	Horse	3.71	0.125	14.7	50	2	15	equCab2	12,000,000	0.26	85.10	34.40	96.32	45.21	45.35	47.01
BEST	JKFC29	PH158	Horse	29.70	1.000	13.7	50	2	14	equCab2	11,000,000	0.50	98.70	40.33	97.60	109.56	8.41	5.31
	JKFC30	PH158	Horse	14.85	0.500	14.7	50	2	15	equCab2	11,000,000	0.37	98.94	40.08	96.32	114.54	8.09	4.97
	JKFC31	PH158	Horse	7.43	0.250	15.8	50	2	16	equCab2	11,000,000	0.34	98.86	38.80	94.37	111.43	7.90	5.10
	JKFC32	PH158	Horse	3.71	0.125	17.2	50	2	17	equCab2	11,000,000	0.18	99.06	38.83	88.59	111.43	7.58	4.88
	JKFC33	PH158	Horse	1.86	0.063	18.4	50	2	18	equCab2	11,000,000	0.07	99.22	38.82	78.31	112.65	7.05	4.72
	JKFC34	PH158	Horse	0.93	0.031	19.9	50	2	20	equCab2	11,000,000	0.03	99.26	39.51	58.17	109.95	6.49	4.37
	JKFC36	PH161	Horse	3.71	0.125	16.4	50	2	16	equCab2	13,000,000	0.03	97.72	49.34	92.90	81.43	13.84	11.24
	JKFC38	PH164	Horse	3.71	0.125	19.9	50	2	20	equCab2	7,200,000	2.02	96.61	0.91	51.28	115.01	20.36	12.39
	JKFC40	MS071	Bison	3.71	0.125	17.4	50	2	17	bison_umd1.0	12,000,000	0.05	96.25	47.95	91.89	58.33	17.54	15.79
	JKFC42	AV005	Horse	3.71	0.125	17.8	50	2	18	equCab2	12,000,000	0.05	95.85	19.63	88.02	48.80	35.71	33.10

Table S3. Summary of library sequencing statistics.
